# 

**Published:** 1906-05-26

**Authors:** 


					TEE HOSPITAL. iTLJWT May 26th 1906.
TAL
1RMCU HOSStlAt ?-
-? ??1 1*~LM*uLascoiy Western Infirmary
No. 1,026? Vol. XL. iSSI!] Satpbdat,
May 26th, 1906.
[Sabfeep!Kn8Terms] PRICE THREEPENCE

				

